# Regulation of Cyclooxygenase-2 Expression by Heat: A Novel Aspect of Heat Shock Factor 1 Function in Human Cells

**DOI:** 10.1371/journal.pone.0031304

**Published:** 2012-02-08

**Authors:** Antonio Rossi, Marta Coccia, Edoardo Trotta, Mara Angelini, M. Gabriella Santoro

**Affiliations:** 1 Institute of Translational Pharmacology, CNR, Rome, Italy; 2 Department of Biology, University of Rome Tor Vergata, Rome, Italy; University Medical Center - University of Groningen, The Netherlands

## Abstract

The heat-shock response, a fundamental defense mechanism against proteotoxic stress, is regulated by a family of heat-shock transcription factors (HSF). In humans HSF1 is considered the central regulator of heat-induced transcriptional responses. The main targets for HSF1 are specific promoter elements (HSE) located upstream of heat-shock genes encoding cytoprotective heat-shock proteins (HSP) with chaperone function. In addition to its cytoprotective function, HSF1 was recently hypothesized to play a more complex role, regulating the expression of non-HSP genes; however, the non-canonical role of HSF1 is still poorly understood. Herein we report that heat-stress promotes the expression of cyclooxygenase-2 (COX-2), a key regulator of inflammation controlling prostanoid and thromboxane synthesis, resulting in the production of high levels of prostaglandin-E_2_ in human cells. We show that heat-induced COX-2 expression is regulated at the transcriptional level via HSF1-mediated signaling and identify, by *in-vitro* reporter gene activity assay and deletion-mutant constructs analysis, the COX-2 heat-responsive promoter region and a new distal *cis*-acting HSE located at position −2495 from the transcription start site. As shown by ChIP analysis, HSF1 is recruited to the COX-2 promoter rapidly after heat treatment; by using shRNA-mediated HSF1 suppression and HSE-deletion from the COX-2 promoter, we demonstrate that HSF1 plays a central role in the transcriptional control of COX-2 by heat. Finally, COX-2 transcription is also induced at febrile temperatures in endothelial cells, suggesting that HSF1-dependent COX-2 expression could contribute to increasing blood prostaglandin levels during fever. The results identify COX-2 as a human non-classical heat-responsive gene, unveiling a new aspect of HSF1 function.

## Introduction

The heat shock response (HSR) is a finely regulated and highly conserved mechanism, which protects living cells against proteotoxic stress induced by different types of environmental and pathological conditions, initiating a regulatory cascade for recovery and adaptation [1]. This occurs by a nearly instantaneous induction of a set of genes, known as heat shock (HS) genes, leading to expression of cytoprotective heat shock proteins (HSP), which is proportional to the intensity and duration of stress [2], [3]. Studies on the heat shock response have revealed insights on the stress-sensing cellular devices and on the role of heat shock proteins in repairing protein damage.

The HSR is regulated by a family of heat shock transcription factors (HSFs) that are expressed and maintained in an inactive state under non-stress conditions. Mammalian genomes encode three homologues of HSF (HSF1, HSF2 and HSF4) regulating HSP expression. Among these HSF1 is considered to be the paralog responsible for regulating the heat-induced transcriptional response [4]. HSF2 has also been reported to contribute to inducible expression of heat shock genes through interplay with HSF1 [5].

HSF1 is generally found in the cytoplasm as an inert monomer lacking transcriptional activity; both DNA-binding and transcriptional transactivation domains are repressed through intramolecular interactions and constitutive serine phosphorylation [6]. Upon exposure to heat shock and other types of stresses, which cause protein damage, HSF1 is derepressed in a stepwise process that involves oligomerization of HSF1 monomers to a trimeric state, localization to the nucleus, inducible phosphorylation and sumoylation, and binding of nuclear-localized trimers to DNA sequences known as heat shock elements (HSE). Functional HSE sequences are characterized by an array of inverted repeats of the pentameric motif nGAAn and are usually located in the proximal region of HSF1-responsive gene promoters.

Binding of HSF1 to HSE sequences is followed by a rapid shift in the transcriptional program resulting in the high rates of transcription of cytoprotective heat shock genes, which include molecular chaperones of the HSP70 and HSP90 families, HSP27 and other proteins of the network. High rates of transcription are maintained as long as HSF1 trimers remain bound to the HSE; when either the stress signal is removed or damaged proteins are no longer generated, the HSR attenuates rapidly, with subsequent conversion of HSF1 back to the monomeric state [7]. Inducible acetylation has also been recently shown to negatively regulate DNA binding activity [8].

Although HSF1 was originally identified as the master regulator of HSP genes, recent studies have indicated an expanding role of this factor, providing evidence that, in some cases, HSF1 may also control the expression of genes with non-chaperone function, some of which participate in the regulation of inflammatory and immune responses [9], [10], [11]. Based on these observations, we investigated the effect of heat stress on the expression of cyclooxygenase 2 (COX-2), a key enzyme in the regulation of the inflammatory response, catalyzing the rate-limiting step in the synthesis of prostanoids and thromboxanes [12], [13]. Herein we report that in human cells heat stress induces the expression of COX-2 at the transcriptional level, by a mechanism involving HSF1-binding to a distal *cis*-acting HSE regulatory sequence located in the promoter at position −2495 from the transcription start site.

## Results

### Heat stress induces the expression of the COX-2 gene in human cells

**Figure 1:**
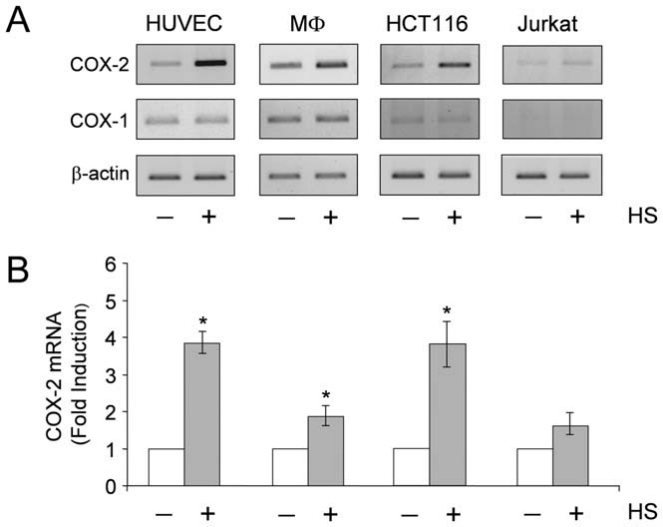
Heat stress induces COX-2 mRNA expression in human cells. Human endothelial cells (HUVEC), peripheral blood monocytes (MΦ), colon carcinoma cells (HCT116) and T lymphoblastoid cells (Jurkat) were either kept at 37°C (- HS) or incubated at 43°C for 40 min (+ HS) and allowed to recover at 37°C for 90 min. At this time, total RNA was extracted and levels of COX-2, COX-1 and β-actin mRNA were analyzed by RT-PCR (**A**) and real-time PCR (**B**). For real-time PCR, relative quantities of COX-2 RNAs were normalized to β-actin. All reactions were made in duplicates using samples derived from at least three biological repeats. Error bars indicate ± S.D. Fold induction was calculated by comparing the induction of the indicated genes in the treated samples to the relative control, which was arbitrarily set to 1. * = P<0.05.

To investigate the effect of heat stress (HS) on COX-2 expression, primary blood monocytes from healthy donors, human umbilical vein endothelial cells (HUVECs) and two human cancer cell lines, HCT116 colorectal adenocarcinoma and lymphoblastoid Jurkat cells, known to express low levels of COX-2 mRNA and protein [14], [15], [16], [17], were incubated at 43°C for 40 min and allowed to recover at 37°C for 90 min. At this time, total RNA was extracted and levels of COX-2, COX-1 and β-actin mRNA were analyzed by RT-PCR and real-time PCR. As shown in Fig. 1A and B, heat treatment resulted in an increase of COX-2 mRNA levels in all cells analyzed; however, HUVECs and HCT116 cells were found to be more sensitive to COX-2 induction by heat as compared to human monocytes and Jurkat cells. Differently from COX-2, no changes in COX-1 levels were detected in the same samples (Fig. 1A). As expected HS also caused an increase in HSP70 mRNA levels in all cell types (data not shown). The elevated COX-2 mRNA levels found in heat-stressed endothelial cells, together with the recent observation that thermal stress may influence endothelium functions [18], prompted us to analyze the mechanism of COX-2 induction by heat in the endothelium.

### Induction of COX-2 expression and PGE production by heat stress in endothelial cells

**Figure 2:**
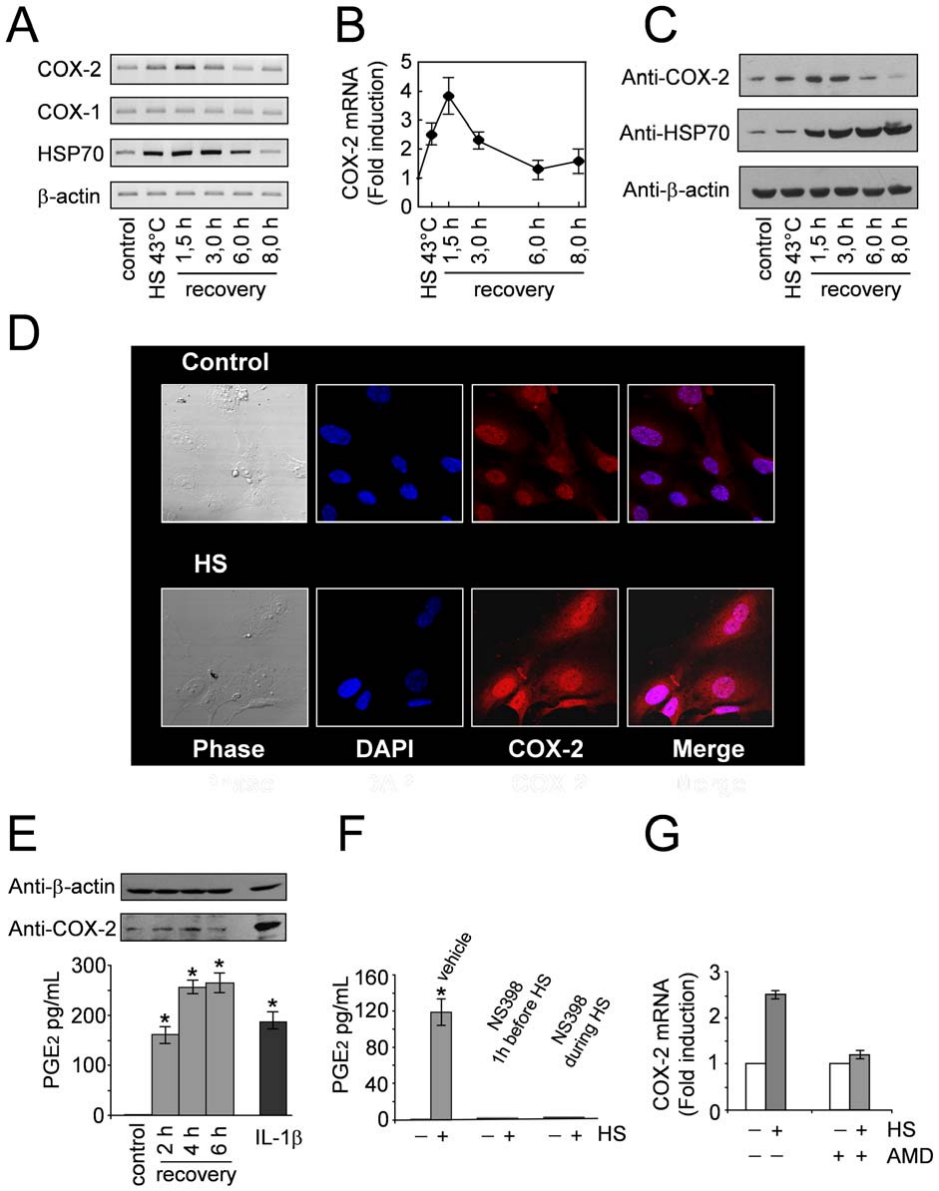
Kinetics of heat-induced COX-2 expression and PGE production in endothelial cells. **A–C.** HUVECs were either kept at 37°C (Control) or were subjected to heat shock (HS) at 43°C for 40 min. At the end of heat treatment, or at 1.5, 3, 6 and 8 hours during the recovery period at 37°C, total RNA was analyzed for COX-2, COX-1, HSP70 and β-actin mRNA by RT-PCR (**A**) and real-time PCR (**B**). In parallel samples whole-cell extracts were analyzed for levels of COX-2, HSP70 and β-actin proteins by Western blot (**C**). (**D**) Immunofluorescence of HUVECs kept at 37°C (Control) or subjected to heat shock at 43°C for 40 min and allowed to recover at 37°C for 3 hours (HS) labeled with anti-COX-2 antibodies (*red*). Nuclei are stained with DAPI (*blue*). The overlay of the two fluorochromes is shown (*Merge*). Images were captured with a Leica confocal microscope TCS 4D system. (**E**) HUVECs were either kept at 37°C (Control) or were subjected to heat shock (HS) at 43°C for 40 min. At 2, 4 and 6 hours during the recovery period at 37°C, whole-cell extracts were analyzed for levels of COX-2 and β-actin proteins by Western blot (top panel), and PGE_2_ production in the culture supernatants was determined by ELISA (bottom panel). In parallel samples treated with IL-1β (20 ng/ml) COX-2 levels and PGE_2_ production were determined 6 hours after treatment. (**F**) HUVECs were treated with the COX-2 inhibitor NS398 (100 µM) or DMSO vehicle either 1 hour before or during HS at 43°C for 40 min. At 2 hours during the recovery period at 37°C, PGE_2_ production was determined by ELISA. Data in **E** and **F** represent the mean±S.D. of triplicate samples from a representative experiment of three with similar results. * = P<0.01. (**G**) HUVECs were treated with actinomycin D (AMD) (5 µg/ml) or vehicle and, after 45 min, were either subjected to heat shock (HS) at 43°C for 40 min or left untreated. At 1,5 hour during the recovery period at 37°C, total RNA was extracted and levels of COX-2 and β-actin mRNA were analyzed by real-time PCR. For real-time PCR (panels **B** and **G**), relative quantities of COX-2 RNAs were normalized to β-actin. All reactions were made in duplicates using samples derived from at least three biological repeats. Error bars indicate ± S.D. Fold induction was calculated as described in Fig. 1.

To gain further insight into the regulation of COX-2 induction in response to heat stress we initially performed kinetics studies on COX-2 mRNA and protein expression in endothelial cells. HUVECs were either kept at 37°C or were subjected to HS at 43°C for 40 min. At the end of heat treatment, or at different times during the recovery period at 37°C, total RNA was analyzed for COX-2, COX-1, HSP70 and β-actin mRNA levels by RT-PCR and real-time PCR. In parallel samples whole-cell extracts were analyzed for levels of COX-2, HSP70 and β-actin proteins by Western blot. As shown in Fig. 2A and B, COX-2 mRNA levels started to accumulate at the end of heat treatment, reaching maximal levels at 1,5 hour during the recovery period, and went back to control levels after 6 hours. No change in COX-1 mRNA levels were observed at all times, while HSP70 mRNA started to accumulate at the end of heat treatment and went back to control levels after 8 hours of recovery at 37°C. Parallel with COX-2 mRNA, COX-2 protein levels started to increase at the end of heat treatment, reached maximal levels at 1,5-3 hours during the recovery period and went back to control levels after 6 hours at 37°C. This is expected since COX-2 protein is typically present for only a few hours after its synthesis, due to the presence of an instability element located upstream of the C-terminus that targets the protein to the ER-associated degradation system and the proteasome [19]. HSP70 protein started to accumulate at the end of heat treatment, but, differently from COX-2, continued to accumulate for at least 8 hours after HS.

The subcellular localization of COX-2 in heat-stressed endothelial cells was examined by confocal microscopy. HUVECs growing on coverslips were subjected to heat stress as described above and allow to recover at 37° for 3 h. As shown in Fig. 2D, under normal conditions COX-2 protein was found to be expressed at low levels and to be localized mainly within the nucleus of HUVECs, as previously reported [20]. In heat-stressed cells, a strong nuclear COX-2 immunofluorescence signal was detected at 3 h after treatment; in addition, increased COX-2 levels were found to be diffusely distributed throughout the cytoplasm (Fig. 2D).

To determine whether COX-2 up-regulation resulted in an enhancement of COX-2 metabolites generation, the production of prostaglandin E_2_ (PGE_2_), a major COX-2 metabolite, was determined. HUVECs were heat stressed as described above and, at different times during the recovery period, whole-cell extracts were analyzed for levels of COX-2 by Western blot, and PGE_2_ production was determined in the culture supernatants by ELISA. As positive control, HUVECs were stimulated with IL-1β and COX-2 levels and PGE_2_ production were determined 6 hours after treatment. As shown in Fig. 2E, heat stress induced a time-dependent increase in PGE_2_ production. As early as 2 hours during the recovery period the increase in PGE_2_ production was comparable to that reached in HUVECs stimulated with IL-1β for 6 hours, despite the fact that higher levels of COX-2 were detected in IL-1β-treated cells. PGE_2_ levels continued to rise until 4–6 hours after stress. Levels of the prostacyclin metabolite 6-keto PGF1α were also found to be increased in the same samples (Rossi A., personal communication).

To determine whether heat-induced PGE_2_ production was dependent on *de-novo* synthesis of heat-induced COX-2 expression or was the consequence of increased prostanoid secretion, HUVECs were treated with the COX-2-selective inhibitor NS398 or DMSO vehicle either 1 hour before or during HS at 43°C for 40 min. After a 2 hour recovery period at 37°C, PGE_2_ production was determined by ELISA. As shown in Fig. 2F, treatment with NS398 resulted in a complete block of PGE_2_ production, demonstrating that heat-induced COX-2 is responsible for prostanoid synthesis during heat stress.

Both mRNA *de novo* transcription and increased mRNA stability have been reported to be involved in the induction of COX-2 [21], [22], [23]. To determine whether gene transcription was required for COX-2 induction by heat, HUVECs were pretreated with actinomycin D (5 µg/ml) for 45 min and then subjected to HS at 43°C for 40 min. At 1,5 hours during the recovery period at 37°C, total RNA was extracted and levels of COX-2 and β-actin mRNA were analyzed by real-time PCR. As shown in Fig. 2G, the increases in COX-2 mRNA level was abolished by actinomycin D treatment, indicating that COX-2 mRNA induction by heat involves *de novo* gene transcription.

### Identification of the promoter elements responsible for COX-2 gene regulation by heat

**Figure 3:**
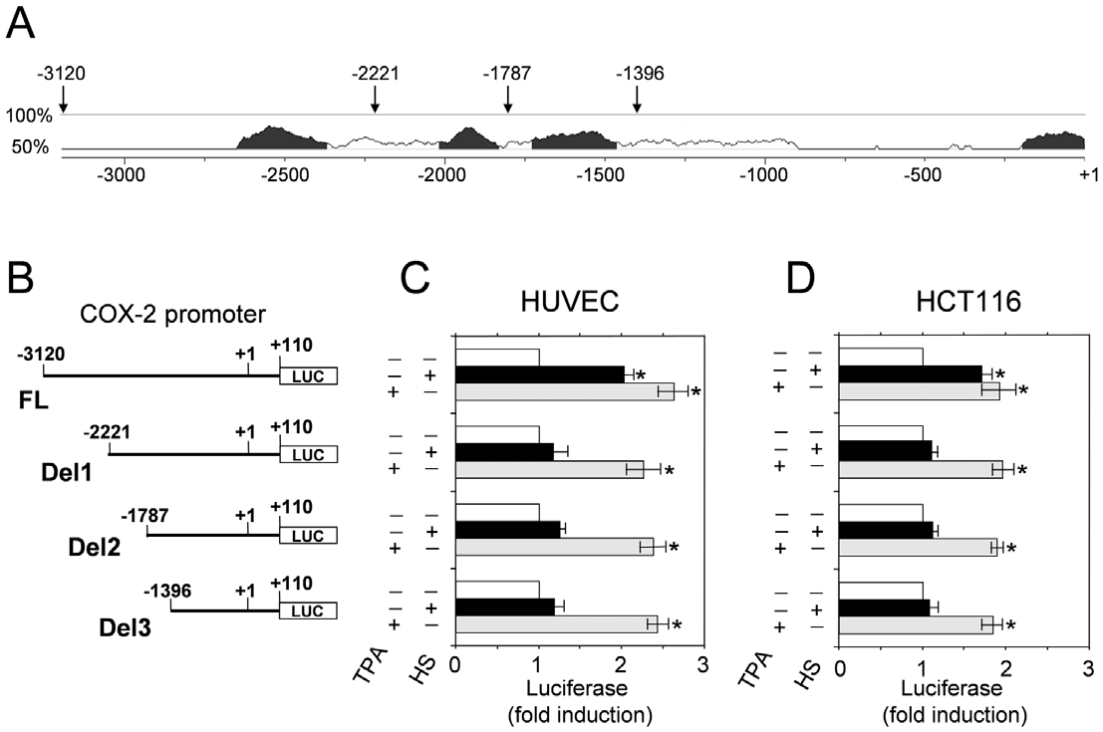
Identification of the promoter region responsible for COX-2 gene regulation by heat. (**A**) Schematic representation of human-mouse conserved sequences in the upstream COX-2 gene promoter region identified by using ECR Evolutionary Conserved Regions (ECR) browser (http://ecrbrowser.dcode.org/). X-axis represents base position from transcription start site (+1), and y-axis indicates percentage identity in a window of 100 nucleotides centered on that position. Black areas represent conserved regions that meet ECR default criteria (ECR similarity ≥70%, ECR length ≥100 nucleotide). The arrows indicate the nucleotide coordinates of the full length and deletion constructs (Del1, Del2 and Del3) represented in **B**. (**B**) Schematic representation of the full-length (FL) and three deletion constructs containing different portions of the 5′-flanking sequences of the human COX-2 promoter region (Del1, Del2 and Del3). The negative numbers (-) noted in each construct indicate the distance from the transcription start site. (**C,D**) Full-length and deletion constructs subcloned into the PGL3-Basic Vector upstream from the luciferase gene were transfected in HUVEC (**C**) and HCT116 (**D**) cells. After 24 h cells were subjected to heat shock (43°C for 40 min) (filled bars), treated with 25 ng/ml TPA (dashed bars) or left untreated (empty bars). Whole-cell extracts were analyzed for luciferase activity after 6 h recovery at 37°C. The data, expressed as fold induction of untreated control, represent the mean of quadruplicate samples from two independent experiments. Error bars indicate ± S.D. * = P<0.05.

The regulation of COX-2 gene (Ptgs2) transcription is very complex, involving distinct signaling pathways and different controlling mechanisms depending on the specific stimulus and the cell type. Sequence analysis of the 5′-flanking region of the human COX-2 gene has identified several potential transcriptional *cis*-acting regulatory elements, located in the proximal region of the COX-promoter within the first 550 bp upstream of the transcriptional start site (TSS), with the exception of a peroxisome proliferator response element (PPRE), that is located at −3721 to −3707 upstream of TSS [22]. To identify novel heat-regulated elements in the COX-2 promoter, we performed a computer-based sequence analysis of the 5′-flanking region. As shown in Fig. 3A, three regions highly conserved in human and mouse were identified between positions −3120 and −1396 upstream of the TSS.

To investigate the role of these regions in COX-2 regulation by heat, a series of 5′-deletion constructs including all three (FL-COX2-PGL3, FL), two (-2221-COX2-PGL3, Del1), one (-1787-COX2-PGL3, Del2) or none (-1396-COX2-PGL3, Del3) of the highly conserved regions were subcloned into the pGL3 reporter vector and used for functional analysis (Fig. 3B). HUVECs and HCT116 cells were transiently transfected with the different constructs; after 24 h, transfected cells were either subjected to heat stress at 43°C for 40 min or stimulated with the COX-2 inducer TPA (12-O**-**tetradecanoylphorbol 13-acetate) as positive control. Luciferase activity was determined after a 6 h recovery period at 37°C. As shown in Fig. 3C and D, heat stress induced reporter activity in both HUVEC or HCT116 cells transfected with the FL construct, while no significant transcriptional activity was detected in cells transfected with Del1, Del2 or Del3 constructs, suggesting that the region spanning between −3120 to −2221 may contain the element/elements responsible for heat-induced COX-2 transcriptional activity. Differently from heat stress, TPA was able to induce significant reporter activity in cells transfected with all four constructs. These results are consistent with the findings of others who identified the elements responsive to TPA in the first 550 bp upstream the TSS [22].

**Figure 4:**
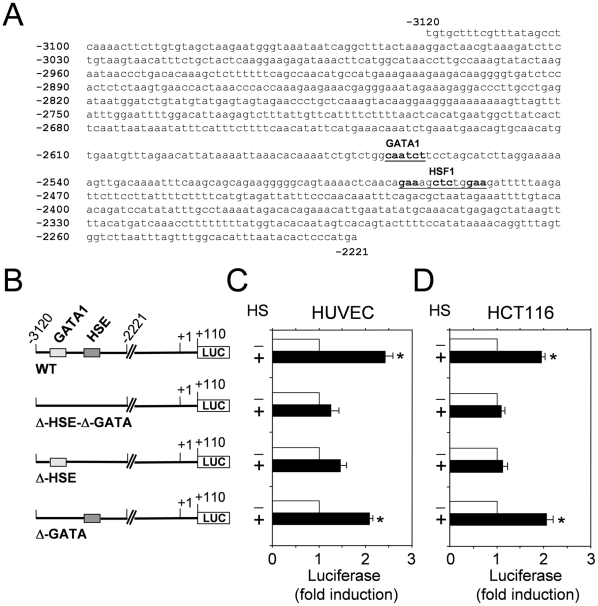
Characterization of distal *cis*-acting HSE regulatory elements in the human COX-2 gene. (**A**) Nucleotide sequence of human COX-2 promoter region between −3120 and −2221, as indicated in Fig. 3A. Location of putative binding sites for HSF1 and GATA transcription factors in the COX-2 promoter identified by TFSEARCH are indicated. (**B**) Schematic representation of the wild type COX-2-PGL3 construct (WT) and the Δ-HSE-Δ-GATA, Δ-HSE and Δ-GATA PGL3 constructs. (**C,D**) WT, Δ-HSE-Δ-GATA, Δ-HSE and Δ-GATA deletion constructs (shown in **B**) were transfected in HUVEC (**C**) and HCT116 (**D**) cells. After 24 h cells were subjected to heat shock (43°C for 40 min) (+ HS), or left untreated (- HS). Whole-cell extracts were analyzed for luciferase activity after 6 h recovery at 37°C. The data, expressed as fold induction of untreated control, represent the mean of quadruplicate samples from two independent experiments. Error bars indicate ± S.D. * = P<0.05.

To identify the enhancer elements responsible for HS-induced COX-2 gene expression, the identified region spanning between −3120 to −2221 was analyzed by TFSEARCH version 1.3 (Yutaka Akiyama, TFSEARCH: Searching Transcription Factor Binding Sites, http://www.cbrc.jp/research/db/FSEARCH.html) [24]. Using a cut-off threshold score of 85%, TFSEARCH prediction analysis identified several transcription factors that may bind to this region. Among these factors, one putative HSF1-binding element located at position −2495 (HSE) and one putative GATA-binding element (GATA-RE) located at −2570 from the transcription start site were detected (Fig. 4A). Since HSEs represent the target for HSF1-mediated heat shock response regulation and GATA response elements were reported to be able to transactivate the HSP70 promoter [25], we focused our attention on these two elements by performing fine structure deletion analysis. A series of constructs deleted for the binding elements for HSF1 (Δ-HSE), GATA1 (Δ-GATA), and for both HSF1 and GATA1 (Δ-HSE-Δ-GATA) were therefore generated (Fig. 4B).

The Δ-HSE-Δ-GATA, Δ-HSE and Δ-GATA or the wild-type (WT) constructs were transiently transfected in HUVEC and HCT116 cells. At 24 h after transfection, cells were subjected to heat stress, and the promoter activity was determined as described above. As shown in Fig. 4C and D, the deletion of both HSE and GATA response elements resulted in an almost complete block of heat-induced transcription. Deletion of the GATA element alone did not alter the ability of the COX-2 promoter to respond to heat; on the other hand, deletion of the HSE element prevented transcriptional activation, identifying the HSE sequence present in the COX-2 promoter as a critical element for heat-induced COX-2 transcription.

### HSF1 binds directly to the COX-2 promoter *in vitro* and *in vivo*

The identification of a functional HSE element in the COX-2 promoter suggested that HSF1 might participate directly in the regulation of COX-2 expression.

**Figure 5:**
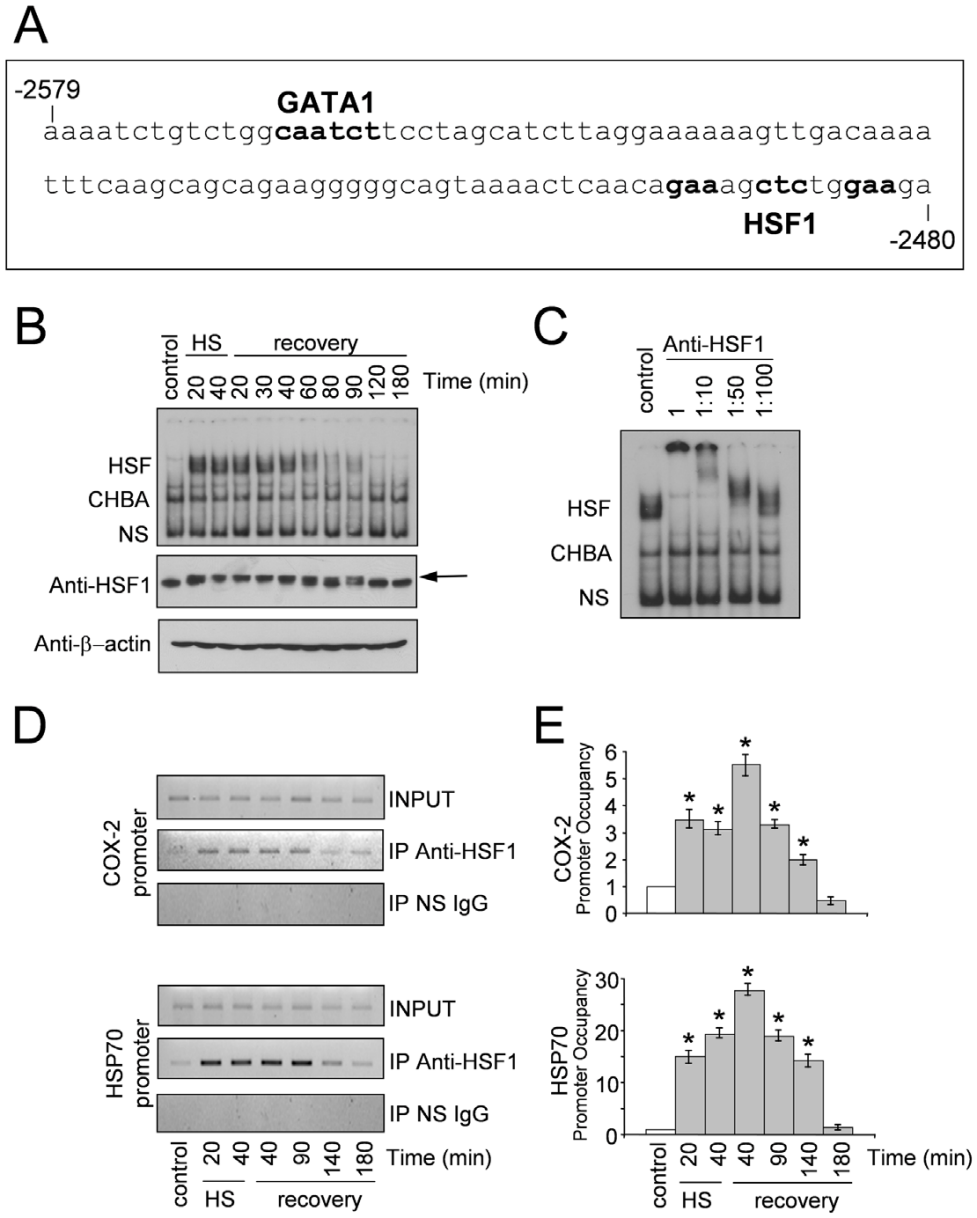
*In vitro* and *in vivo* binding of HSF1 to the COX-2 promoter. (**A**) Nucleotide sequence of the 100 bp (from −2579 to −2480) DNA fragment of the COX-2 promoter region including binding sites for the GATA and HSF1 transcription factors. The 100 bp fragment was amplified by PCR, ^32^P-labeled and used as probe for gel-shift analysis shown in **B** and **C**. (**B**) HUVECs were subjected to heat shock at 43°C or left untreated (control). After 20 and 40 min at 43°C (HS) or at the indicated times during recovery at 37°C (recovery), whole-cell extracts were analyzed for HSF DNA-binding activity by EMSA in a 3% polyacrylamide gel using the probe described in (**A**). Position of HSF-DNA binding complex (HSF), constitutive HSE binding activity (CHBA) and non specific protein-DNA interactions (NS) are shown (upper panel). In parallel samples whole-cell extracts were analyzed for levels of HSF1 and β-actin proteins by Western blot (lower panels). Arrow indicates the position of the low-mobility phosphorylated HSF1 isoform. (**C**) Specificity of HSF1-DNA binding complexes. Whole-cell extracts from HUVECs subjected to heat shock at 43°C for 40 min were preincubated with different dilutions of anti-HSF1 polyclonal antibodies for 15 min before supershift assay. Position of HSF, CHBA and NS are indicated as in **B**. (**D,E**) HUVECs were subjected to heat shock at 43°C or left untreated (control). After 20 and 40 min at 43°C (HS) or at the indicated times during recovery at 37°C (recovery), recruitment of HSF1 to the COX-2 and HSP70 promoters was analyzed by ChIP assay. (**D**) ChIP-enriched DNAs using preimmune serum (IP NS IgG) or anti-HSF1 serum (IP anti-HSF1), as well as input DNAs (INPUT) were prepared, and DNA fragments of the COX-2 gene (−2629 to −2420) and HSP70 gene (−262 to −70) were amplified by PCR. (**E**) Quantification of ChIP assay shown in (**D**). Samples from at least three independent experiments were analyzed by real time PCR. Relative promoter occupancy is expressed as fold induction of control arbitrarily set to a value of 1. Error bars indicate ± S.D. * = P<0.05.

Heat stress is known to activate HSF1 as early as few minutes. To establish the role of HSF1 in COX-2 up-regulation by heat, kinetics experiments were performed to determine HSF1 DNA-binding activity to the COX-2 promoter *in vitro* and *in vivo*. HUVECs were subjected to a 40 min heat shock at 43°C and then allowed to recover at 37°C. At different times during heat treatment or the recovery period, whole-cell extracts were analyzed for HSF1 DNA-binding activity by EMSA using a large DNA fragment of 100 bp (from −2579 to −2480) of the COX-2 promoter region comprising binding sites for the GATA and HSF1 transcription factors (Fig. 5A). In parallel, the same samples were analyzed for HSF1 DNA-binding activity by EMSA using a 30 bp oligonucleotide containing an ideal HSE from the HSP70 promoter [26].

A specific slower-migrating mobility complex, subsequently identified as HSF1 (see below), was found to appear as early as 20 min in the heat-shocked samples using the COX-2 promoter derived 100 bp sequence (Fig. 5B, upper panel). The slower-migrating mobility complex persisted up to 40 min of the recovery period, after which time it started to attenuate. This kinetics is consistent with the kinetics of HSF1 phosphorylation/dephosphorylation, which is known to be strictly associated with HSF1-DNA binding activity (Fig. 5B middle panel) [27]. A similar DNA-binding pattern was obtained when the ideal HSE probe was used (Fig. S1). Similarly to the DNA-binding analysis using the ideal HSE element, two faster migrating bands present in both heat-treated and control samples representing the non-specific (NS) and the constitutive HSE binding activity (CHBA) respectively [26], were detected also using the COX-2 promoter derived sequence.

To determine whether HSF1 was involved in the formation of the slower-migrating mobility complex observed in Fig. 5B, a super-shift analysis was performed using specific polyclonal anti-HSF1 antibodies. As shown in Fig. 5C, the complex was selectively super-shifted in the presence of anti-HSF1 antibodies, indicating that HSF1 is the primary component of the heat-induced DNA binding activity observed.

To investigate whether HSF1 is recruited to the COX-2 gene promoter, HUVECs were subjected to a 40 min heat shock at 43°C and then allowed to recover at 37°C. At different times during heat treatment and the recovery period HSF1 recruitment to the COX-2 and HSP70 promoters was analyzed by ChIP analysis. HSF1-coprecipitating DNA was analyzed by PCR (Fig. 5D) and real-time PCR (Fig. 5E) with promoter-specific primers amplifying the COX-2 and HSP70 promoters respectively, and the rate of amplification was verified using cross-linked, not immunoprecipitated chromatin. The specificity of chromatin immunoprecipitation was determined by using a control unrelated antibody. Consistently with the DNA-binding activity revealed by EMSA (Fig. 5B, upper panel), HSF1 was recruited to the COX-2 promoter during heat treatment and for at least 90 min of the recovery period at 37°C (Fig. 5D,E upper panels). A similar kinetics of HSF1 recruitment was observed also for the HSP70 promoter (Fig. 5D,E lower panels).

An identical set of experiments was performed in HCT116 cells. As shown in Fig. S2, the results obtained were comparable to the ones described above for HUVECs. In addition, heat-induced COX-2 mRNA expression was greatly reduced in HCT116 cells in which HSF1 expression was stably suppressed by RNA interference (Fig. S3), confirming an important role of this factor in heat-regulated COX-2 transcription.

### COX-2 expression is induced at febrile temperatures in endothelial cells

**Figure 6:**
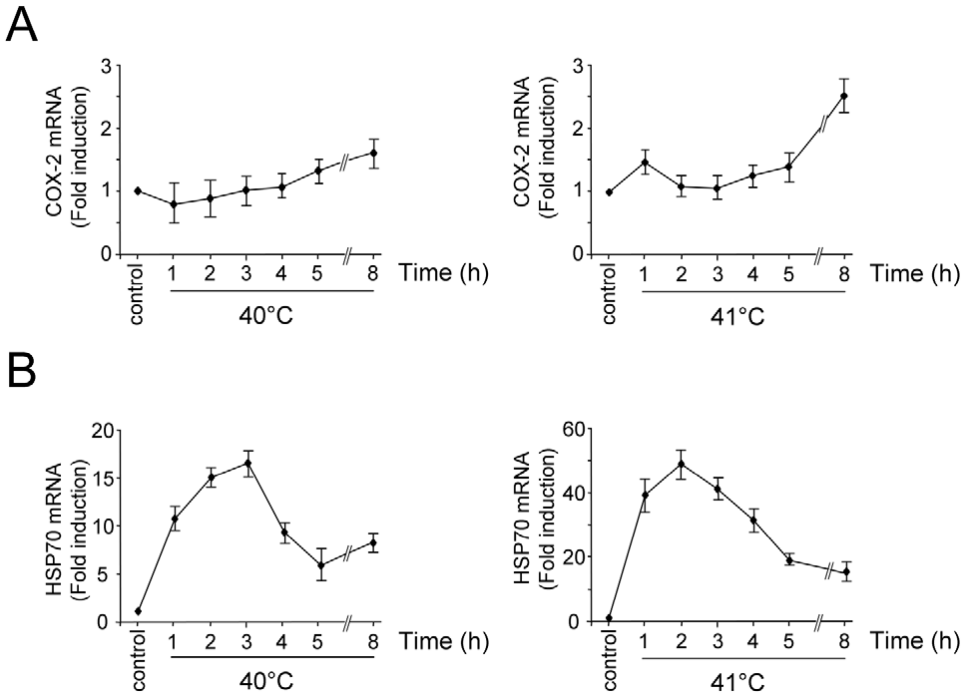
Time-course of COX-2 expression at febrile temperatures in endothelial cells. HUVECs were either kept at 37°C (control) or were incubated at 40 and 41°C as described in Material and Methods. At different times total RNA was extracted and levels of COX-2 (**A**) and HSP70 (**B**) were analyzed by real-time PCR. Relative quantities of COX-2 and HSP70 RNAs were normalized to β-actin. All reactions were made in duplicates using samples derived from at least three biological repeats. Error bars indicate ± S.D. Fold induction was calculated by comparing the induction of the indicated genes in the treated samples to the relative control, which was arbitrarily set to 1.

Partial activation of HSF1 has been described during exposure to febrile, sub-heat shock temperatures [28], [29], [30], [31]. To investigate whether COX-2 expression may be induced at febrile temperatures in endothelial cells, HUVECs were either kept at 37°C or were incubated at 40 and 41°C for 8 hours. At different times after treatment total RNA was extracted and levels of COX-2, HSP70 and β-actin as control were analyzed by real-time PCR. As shown in Fig. 6A, COX-2 expression was increased, reaching maximal levels at 8 hours of continuous exposure to physiological fever temperature. At this time a modest increase in COX-2 protein level and PGE_2_ production was also observed (Fig. S4); however, the effect of febrile temperatures on the kinetics of different COX-2 metabolites production needs to be further investigated. Interestingly, the kinetics of heat-induced COX-2 expression differed from HSP70 mRNA accumulation, that, as expected, reached a pick at 2–3 hours after exposure (Fig. 6B).

## Discussion

Cyclooxygenases-1 and -2 (COX-1 and COX-2), also known as prostaglandin (PG) endoperoxide synthase, are heme-containing ER membrane-bound N-glycoproteins that catalyze the committed step in prostaglandin formation. COX-1 and −2 convert arachidonic acid, hydrolyzed from cell membrane phospholipids by a phospholipase A2 (PLA-2), to prostaglandin endoperoxide H2 (PGH2), the precursor of thromboxanes, prostacyclin and different prostaglandins [23], [32]. COX-1 is constitutively expressed in almost all mammalian tissues under basal conditions, while COX-2 expression is highly restricted (except for kidneys, seminal vesicles and certain areas of the brain), but can be dramatically upregulated following the appropriate stimuli [22]. In addition, differences in regulation of the COX enzymes at the post-transcriptional and post-translational levels also contribute significantly to their distinct patterns of expression. COX-1 mRNA and COX-1 protein are very stable, whereas both COX-2 mRNA and COX-2 protein have short half-lives. The 3′-UTR of the human COX-2 gene contains 23 copies of the ‘ATTTA’ RNA instability element that participates in post-transcriptional regulation of COX-2 expression [33]. On the other hand, a unique 27 amino acid instability element located upstream of the C-terminus of COX-2 targets this isoform to the ER-associated degradation system and proteolysis by the cytosolic 26 S proteasome [19]. Therefore, COX-2 protein is typically present for only a few hours after its synthesis.

Depending on the cell type, COX-2 expression can be rapidly induced by a large number of stimuli, which include proinflammatory cytokines such as IL-1, IL-2 and TNFα, bacterial endotoxin (LPS), growth factors, oncogenes, and tumor promoters (TPA) [22]. The regulation of COX-2 gene transcription is very complex, involving distinct signaling pathways and different controlling mechanisms depending on the specific stimulus and the cell type. The promoter region of the COX-2 gene presents sequences of typical immediate early genes with potential transcriptional regulatory *cis*-elements mainly located in the 550 nucleotide region upstream of the transcriptional start site (TSS); these include two cyclic AMP response elements (CRE), a sterol response element (SRE), two nuclear factor kappa B (NF-κB) sites, an SP1 site, a CAAT enhancer binding protein C/EBP, two AP-1 sites, an E-box, and a TATA box [22]. A distal peroxisome proliferator response element (PPRE) has also been identified at position −3721 from the TSS [34]. The relative contribution of each *cis*-acting promoter element and its interaction with transcription factors activated by multiple signaling pathways depends on the cell type, the stimulus and the time following the stimulus [22].

We have now identified a new putative distal *cis*-acting heat shock element (HSE) located at position −2495 from the TSS, responsible for binding the heat-regulated factor HSF1.

We provide evidence that heat stress promotes COX-2 expression in human cells, and that this effect is mediated by HSF1. Following heat stress at 43°C for 40 min, COX-2 mRNA levels start to accumulate at the end of heat treatment, reaching maximal levels at 1,5 hour during the recovery period. Similarly to the effect described for other COX-2 inducers, heat-induced COX-2 expression is transient, and COX-2 levels return back to basal 6 hours after heat exposure. No change in COX-1 mRNA levels are observed at all times after HS. Parallel with COX-2 mRNA, COX-2 protein levels were also found to increase at the end of heat treatment, reaching maximal levels at 1,5–3 hours during the recovery period at 37°C. Differently from the heat shock protein HSP70, COX-2 protein returned to basal levels after 6 hours at physiological temperature, possibly due to proteolysis by the 26 S proteasome.

Both mRNA *de novo* transcription and increased mRNA stability have been reported to be involved in the induction of COX-2 [22], [23]. We have shown that heat-induced COX-2 expression is regulated at the transcriptional level and have identified, by *in vitro* reporter gene activity assay and deletion mutant constructs analysis, the promoter region responsive to heat induction and the position of the HSE involved. As indicated above, the new putative *cis*-acting heat shock element identified was located in a distal region of the COX-2 promoter at position −2495 from the transcription start site. *In vitro* and *in vivo* binding of HSF1 to the HSE identified in the COX-2 promoter was demonstrated by gel shift analysis and chromatin immunoprecipitation analysis, respectively. Deletion of the HSE sequence resulted in abrogation of heat-induced COX-2 transcription, indicating that HSF1 plays a central role in the transcriptional control of this gene during heat-stress.

The presence of a distal HSE sequence located at −2495 from the TSS in the COX-2 promoter adds a new element in the complex regulation of COX-2 expression and also represents a novel finding in heat shock regulation of human genes. In fact the functional HSEs characterized so far in the promoters of classical and non-classical heat-shock human genes are usually restricted to the promoter region proximal (<1000 bp) to the TSS [35]. In the case of the lactate dehydrogenase promoter HSF1 binding to a distal HSE located at −3819 from the TSS was described following ERB2 overexpression [36]. However, HSF1 recruitment to distal HSEs leading to transcriptional activation under heat stress conditions has never been described to our knowledge.

As indicated above, COX-2 transcription is dependent on the specific stimulus and the cell type. In response to heat we also observed different levels of COX-2 expression in different human primary and tumor cell lines. COX-2 levels appear to be enhanced considerably (3–4 fold as compared to control) in human endothelial cells and HCT116 colon carcinoma cells, and to a lesser extent in primary monocytes and lymphoblastoid cells (Fig. 1), but not in several breast cancer cell lines (data not shown). This effect could be due to the presence of other cell-type specific transcription factors needed to cooperate with HSF1 in COX-2 induction. On the other hand, it can be speculated that the differences observed in heat-induced COX-2 expression in different cell types may reflect differences in the chromatin modification signature associated with highly cell-type specific histone modification patterns, which in turn can affect HSF1 binding and/or its transcriptional potential [37], [38]. It has been in fact recently demonstrated in *Drosophila* that the chromatin landscape before heat shock can dictate HSF binding to target DNA elements [39]. In the case of classical heat shock genes, however, because of the ubiquitous and conserved nature of the HSR, functional HSE binding sites are likely to be evolutionary constrained at the sequence level and actively maintained in the accessible state at the level of chromatin organization in all cell types [39].

The possibility of interplay of different HSFs in the regulation of COX-2 should be also considered. As indicated in the Introduction, in human cells three different HSF family members exist, which exhibit differential specificity for different types of heat shock elements [5], [40]. This, together with cell type-specific expression of HSFs [5], [40], may be important in determining the target genes of each heat shock factor.

COX-2 up-regulation resulted in an enhancement of COX-2 metabolites generation, as demonstrated by the production of PGE_2_. It should be noted that, despite the moderate increase in COX-2 mRNA level (3–4 fold) as compared to a strong induction of classical heat shock genes like HSP70 (50–80 fold), a potent biological response is achieved. In fact, as early as 2 hours after heat stress, the increase in PGE_2_ production was comparable to that reached in cells stimulated with the COX-2 inducer IL-1β, and PGE_2_ levels continued to rise until 4–6 hours after stress. It should be noted that an increase in arachidonic acid metabolites production, associated to stimulation of a phospholipase A2 (PLA-2) activity, following heat shock has been previously reported in mammalian cells [41]. In the case of endothelial cells, the fact that treatment with the selective COX-2 inhibitor NS398 resulted in a complete block of PGE_2_ production, demonstrates that PGE_2_ production is dependent on *de-novo* synthesis of heat-induced COX-2 and is not merely the consequence of increased prostanoid secretion. This observation, together with the previously described increase of phospholipase A2 activity by heat, suggests the possibility that heat stress may coordinate the entire cascade of prostanoid synthesis by simultaneously inducing COX-2 protein expression and stimulating PLA-2 activity to increase COX-2 substrate bioavailability.

Increased COX-2 levels have been previously observed at elevated temperatures *in vivo* in murine models [42], [43]. The possibility that also in human cells COX-2 expression may be induced *in vivo* at high temperatures, as in the case of burn injuries or hyperthermic treatment of cancer, should be considered. In the case of burns, that are known to cause the release of arachidonic acid metabolites [44], since thermal insults activate an inflammatory cascade which culminates in the infiltration of specialized cells into injury sites, it could be hypothesized that HSF1-induced COX-2 expression may participate in these events during the initial phase of inflammation. Elevated temperatures are also reached during hyperthermic treatment of tumors [45]. In particular, in the case of whole-body hyperthermia, a procedure used for metastatic cancer treatment, temperatures up to 42°C are reached and maintained for about 1 hour in the entire body [46]. In this case, vascular endothelium is subjected to heat stress conditions, which *in vitro* induce COX-2 expression and prostaglandins production. The possibility that COX-2 expression and production of high levels of arachidonic acid metabolites may be induced under these conditions should be taken into consideration, especially in view of treatment with non-steroidal anti-inflammatory drugs.

Interestingly, COX-2 expression was also induced at mild (40 and 41°C) temperatures in endothelial cells, reaching maximal levels after 8 hours of exposure, suggesting the possibility that COX-2 expression could be increased during fever. This phenomenon could contribute to the increase in blood prostaglandin level that has been documented previously in the late phase of fever [47]. The role of HSF1, as well as HSF2, which has been recently shown to be a primary factor required for maintaining protein homeostasis against febrile range thermal stress in murine cells [48], on COX-2 regulation at febrile temperatures needs to be further investigated.

Apart of the possible physiological implications, the identification of COX-2 as an HSF1-target gene opens new perspectives on the role of this factor. HSF1 was originally identified as the master regulator of the transcription of heat shock genes, leading to the rapid expression of cytoprotective heat shock proteins. In mammalian organisms during stress conditions different HSP act in concert constituting the cell “protein repair machinery” preventing protein denaturation and aggregation that are detrimental to cells, and promoting degradation of irreversibly denaturated cytotoxic proteins [3], [49], [50]. Under normal conditions, HSP also function as chaperones that assist in protein folding [51]. Whereas it is well established that HSF1 regulates inducible HSP expression, recently it has become evident that the regulation of the mammalian HSR is a more complex phenomenon than previously thought, and in some cases HSF1 may also control the expression of genes with non-chaperone function, some of which participate in the regulation of the inflammatory and immune responses, including interleukins 1β (IL-1β), 6 (IL6), tumor necrosis factor α (TNFα) and different chemokines [11], [52], [53], [54]. The fact that HSF1 also regulates the expression of an important mediator of inflammation as COX-2 reinforces these previous observations, and brings the attention to this less known and still poorly understood role of HSF1 during thermal stress. It could be hypothesized that if, on one side, HSF1 activates a fundamental and highly conserved defense response through the robust expression of classical heat shock genes with chaperone functions to protect individual cells, on the other it may also coordinate a different type of response to thermal injury, through a more finely tuned, cell-type specific induction of non-chaperone heat-responsive genes, among which COX-2. The understanding of this different aspect of HSF1 function may indicate new strategies in the treatment of immune and inflammatory disorders.

## Materials and Methods

### Cell culture and treatments

Primary human umbilical vein endothelial cells (HUVEC) were purchased from Cambrex Bio Science (Walkersville, MD, USA) and grown in EGM-2 complete medium (Cambrex Bio Science) according to the manufacturer's instructions. Cells were incubated at 37°C with 5% CO_2_ in non-coated 100 mm BD Falcon™ cell culture dishes (BD Biosciences Discovery Labware). Culture medium was changed at day 1 and every 2 days thereafter. Monolayer cell confluence was achieved after 4–6 days of culture. All experiments were performed using HUVEC passage 2–5. Human colorectal adenocarcinoma (HCT116) and lymphoblastoid (Jurkat) cells (American Type Culture Collection) were maintained in McCoy's 5 A (HCT116) or RPMI 1640 (Jurkat) medium supplemented with 10% FCS, 2 mM glutamine, and antibiotics. Human peripheral blood monocytes, isolated and purified from buffy coat of healthy blood donors (kindly provided by Prof. Adorno, Haematology Division, University of Rome Tor Vergata) as described elsewhere [10], were grown for 24 h in RPMI 1640 medium, supplemented with 10% FCS and antibiotics as indicated above. For heating procedures, cells were subjected to heat shock at the indicated temperatures in a precision water bath-W14 (Grant Instruments). For long exposure at febrile temperatures HUVECs were incubated in automatic CO_2_ incubators certified to have <0.2°C temperature variation and calibrated for each experiment using an electronic thermometer. Selective COX-2 inhibitor NS-398, tetradecanoylphorbol 13-acetate (TPA) and interleukin 1β (IL-1β) (Sigma) were dissolved in dimethylsulfoxide (DMSO) and diluted in the culture medium immediately before use. Control cells received the same amount of vehicle.

### Cloning of COX-2 promoter, preparation of 5′ deletion constructs and mutants

To generate the COX-2-PGL3 vector indicated as full-length (FL) in this study, a pair of gene-specific primers (sense: 5′-CGGGCTAGCTGTGCTTTCGTTTATAGCCTCA-3′; antisense: 5′-GCTAAAGCTTGGGTAGGCTTTGCTGTCTGA-3′) was designed to amplify the COX-2 gene promoter region (spanning from −3120 upstream of the gene transcription start site to +110) from human genomic DNA (Promega) by using Phusion High-Fidelity DNA polymerase (Finnzymes). The reaction product was analyzed by agarose gel electrophoresis, digested with *NheI* and *HindIII*, and inserted upstream of the luciferase gene of the pGL3Basic vector (Promega) to generate the FL-COX2-PGL3 (FL) construct. The -2221-COX2-PGL3 (−2221, Del1), -1787-COX2-PGL3 (−1787, Del2) and -1396-COX2-PGL3 (−1396, Del3) serial deletion mutants were generated as described above. Oligos used were: for −2221, Del1 construct, sense: 5′- CGGGCTAGCACCAGCATCCCAAATGTACC-3′; for -1787, Del2 construct, sense: 5′- CGGGCTAGCAACATGGCTTCTAACCCAAA-3′; for −1396, Del3 construct, sense: 5′- CGGGCTAGCGCTGTCATTTTCCTGTAATGC-3′. The antisense oligo used was the same as that used for the construction of the FL construct. The Δ-HSE-COX2-PGL3 (Δ-HSE), Δ-GATA-COX2-PGL3 (Δ-GATA) and Δ-HSE-Δ-GATA-COX2-PGL3 (Δ-HSE-Δ-GATA) mutant constructs were generated by using the QuikChange Site-Directed Mutagenesis Kit following the manufacturer's instructions (Stratagene). Oligos used were: for Δ-HSE construct, sense: 5′-GCAGAAGGGGGCAGTAAAACTAGCTCTGGAAG-3′, antisense: 5′-CTTCCAGAGCTAGTTTTACTGCCCCCTTCTGC-3′; for Δ-GATA construct, sense: 5′-ACACAAAATCTGTCTTCCTAGCATCTTAGGAAAAAAGTTG-3′, antisense: 5′-CAACTTTTTTCCTAAGATGCTAGGAAGACAGATTTTGTGT-3′. For the Δ-HSE-Δ-GATA construct, the same oligos used for Δ-GATA were used on the Δ-HSE construct. The nucleotide sequence of each construct was verified by DNA sequencing.

### Recombinant retroviral vectors

For HSF1 knockdown experiments, we used the RNAi-pSuper-retro vector, containing a puromycin resistance gene for selection of stable transfectants. The sequence of human HSF1 gene was selected as reported before [55]. Retroviruses were produced by transfection of 293T cells with plasmids expressing retroviral proteins Gag-Pol, G (VSV-G pseudotype), pSUPER-retro and HSF1i-pSUPER-retro constructs using Lipofectamine 2000 (Invitrogen). At 48 h after transfection, supernatants containing the retroviral particles were collected and frozen at −70°C until use. HCT116 cells were infected with diluted supernatant in the presence of 8 µg/ml Polybrene overnight, and then selected with puromycin (1 µg/ml) 48 h after infection. After 10 days in selective medium, two pools referred to as pHCT116-pSUPER-retro (pHCT116-retro) and pHCT116-pSUPER-retro-HSF1i (pHCT116-HSFi) were isolated. The puromycin selective pressure was removed 24 hours before experimental procedures.

### Cell transfection and reporter assays

Transfections were performed using FuGENE HD Transfection Reagent (Roche) for HUVEC cells and LipofectAMINE Plus reagent (Invitrogen) for HCT116 cells according to the manufacturer's protocols. For reporter gene experiments, the different COX-2 constructs were co-transfected with a control plasmid (pRL-TK encoding *Renilla* luciferase; Promega) to normalize transfection efficiency. Transfected cells were grown for 16 h before heat treatment and luciferase activity of quadruplicate samples was measured in a Microplate Luminometer (Wallac-Perkin Elmer) using Dual-Luciferase kit (Promega). COX-2 promoter firefly luciferase activity was normalized to *Renilla* luciferase activity in the same sample.

### Electrophoretic Mobility Shift Assay (EMSA)

The 100 bp DNA probe (shown in Fig. 5A) was prepared by PCR amplification of the COX-2 promoter region containing the HSF1 binding site (from −2579 to −2480) using the following primers: sense: 5′- AAAATCTGTCTGGCAATCTTCC-3′, antisense: 5′- TCTTCCAGAGCTTTCTGTTGAG-3′. This PCR product was labeled at both ends using [γ^32^-P]ATP plus T4 nucleotide kinase. Whole-cell or nuclear extracts were prepared as described [56]. Whole-cell (15 µg) and nuclear (5 µg) extracts were incubated with a ^32^P-labeled probe followed by analysis of DNA-binding activity by EMSA. Binding reactions were performed as described [57]. Complexes were analyzed by nondenaturing 3% polyacrylamide gel electrophoresis. Quantitative evaluation of HSF-HSE complex formation was determined by Typhoon 8600 imager (Molecular Dynamics, Amersham Biosciences) with the use of ImageQuant (Amersham Biosciences).

### Western blot analysis

Equal amounts of protein (35 µg/sample) from whole-cell extracts were separated by SDS/PAGE, and blotted to nitrocellulose. After blocking with 5% skim milk solution, membranes were incubated with rabbit polyclonal anti-HSF1, (Santa Cruz Biotechnology), antibodies, or monoclonal anti-COX-2 (SC-19999, Santa Cruz Biotechnology), anti-HSP70 (Stressgene), and anti-β-actin (Sigma) antibodies followed by decoration with peroxidase-labeled anti-rabbit or anti-mouse IgG respectively (Super Signal detection kit, Pierce).

### RNA extraction, RT-PCR and real-time RT-PCR

Total RNA was prepared using Trizol (Invitrogen) as described in the manufacturer's protocol. For RT-PCR analysis, extracted RNA (1 µg) was digested with 2 U of DNase I (Invitrogen) for 30 min at 37°C. Samples were reverse transcribed to cDNA with 200 U of Moloney murine leukemia virus reverse transcriptase (RT) (Gibco-BRL Life Technologies), using 5 µg of random primers (Invitrogen) for 1 h at 45°C in a total volume of 20 µl. RT was inactivated at 95°C for 5 min. For each sample, an aliquot of DNase I-digested RNA, without RT, was used as a negative control for PCR amplification. The sequences of COX-2, COX-1, HSP70 and β-actin primers were as follows: COX-2, sense: 5′- CAGCACTTCACGCATCAGTT-3′, antisense: 5′- CAGCAAACCGTAGATGCTCA-3′; COX-1, sense: 5′- AACATGGACCACCACATCCT-3′, antisense: 5′- TCCAGGGTAGAACTCCAACG -3′; HSP701A, sense: 5′-CTACAAGGGGGAGACCAAGG-3′, antisense: 5′- TTCACCAGCCTGTTGTCAAA-3′; β-actin, sense: 5′-GCGCTCAGGAGGAGCAAT-3′, antisense: 5′-GCACTCTTCCAGCCTTCC-3′. Amplification was performed using the following parameters: 94°C for 45 s, 60°C for 30 s, and 72°C for 45 s for 28 cycles for COX-2, COX-1 and HSP701A, and 24 cycles for β-actin. PCR products were electrophoresed alongside DNA Molecular Weight Marker IX (Roche Applied Science) in 2% agarose gels and then stained with ethidium bromide. PCR amplification was performed in a thermal cycler GeneAmp 2400 (Applied Biosystems), using Hot-Start *Taq* polymerase (QIAGEN). Real-time RT-PCR analysis was performed with ABI PRISM 7000 (Applied Biosystem, USA), using RealMasterMix ROX (Eppendorf) to prepare the reaction mixes. Primers used for real-time PCR of human genes were identical to the primers described above for semiquantitative RT-PCR. Relative quantities of selected mRNAs were normalized to β-actin in the same samples.

### Chromatin Immunoprecipitation (ChIP) assay

Cells were fixed by adding formaldehyde (Sigma) to the medium to a final concentration of 1%. After 15 min, cells were washed with ice-cold phosphate-buffered saline (PBS) containing 1 mM phenylmethylsulfonyl fluoride, shaken for 20 min at 4°C in Lysis Buffer-1 (50 mM HEPES-KOH, pH 7.5, 140 mM NaCl, 1 mM EDTA, 10% glycerol, 0.5% NP-40, 0.25% Triton X-100, containing protease inhibitors) and harvested using a cell scraper. After centrifugation at 3000 rpm for 10 min the pellet was resuspended in Lysis Buffer-2 (10 mM Tris-HCl, pH 8.0, 200 mM NaCl, 1 mM EDTA, 0.5 mM EGTA, containing protease inhibitors) and shaken at room temperature for 10 minutes. After centrifugation, nuclei were resuspended in Lysis Buffer-3 (50 mM Tris, pH 8.0, 1% SDS, 5 mM EDTA), and chromatin was sheared by sonication. After removal of nuclear debris by centrifugation at 13,000 rpm for 5 min at 8°C, lysates were diluted 10-fold with DB buffer (50 mM Tris pH 8.0, 5 mM EDTA, 200 mM NaCl, 0.5% NP40) and then precleared for 3 h using 80 µl of 50% salmon sperm-DNA saturated protein A (ss-proteinA)-agarose beads. Immunoprecipitation was carried out at 4°C overnight, and immune complexes were collected with ss-proteinA-agarose beads. Antibodies utilized included anti-HSF1 (Santa Cruz Biotechnologies) or pre-immune rabbit serum as control for non-specific interaction. After washing three times with high salt WB buffer (20 mM Tris, pH 8.0, 0.1% SDS, 1% NP-40, 2 mM EDTA, 0.5 M NaCl) and twice with low salt TE buffer (10 mM Tris, pH 8.0, 1 mM EDTA), immunocomplexes were eluted with TE containing 1% SDS. Protein-DNA cross-links were reverted by incubating at 65°C overnight. After proteinase K digestion, DNA was extracted with phenol-chloroform and precipitated with ethanol using 15 µg of tRNA as carrier. PCR was performed using the following primers: COX-2, sense: 5′-AAATGAACAGTGCAACATGTGA-3′, antisense: 5′-CGTCTGAAATTTGTTGGGAAA-3′; HSP701A, sense: 5′-CACTCCCCCTTCCTCTCAG-3′, antisense: 5′-TTCCCTTCTGAGCCAATCAC-3′. For quantification of ChIP assays, samples from at least three independent experiments were analyzed by real time PCR. Data were normalized to the input DNA and DNA from untreated cells.

### PGE_2_ measurements

PGE_2_ production in the supernatants of endothelial cells was determined by PGE_2_ EIA kit (Cayman Chemical Co.) according to the manufacturer's instructions. The results are expressed as picograms per milliliter, calculated according to the PGE_2_ standard curve. All assays were performed in triplicate.

### Confocal microscopy

HUVECs grown on coverslips were fixed with 4% paraformaldehyde and permeabilized in 0,1% Triton X-100-PBS for 10 min. After blocking with 0,2% BSA, fixed cells were incubated with rabbit anti-COX-2 antibody (SC-1747 Santa Cruz Biotechnology) for 1 hour, and incubated with cy3-conjugated anti-rabbit IgG for 30 min at room temperature. Coverslips were mounted in Vectashield medium (Vector Laboratories). Images were acquired on a Leica confocal microscope TCS 4D system, equipped with a 100X 1.3–0.6 oil immersion objective.

### Statistical analysis

Statistical analysis was performed using the Student's *t* test for unpaired data. Data are expressed as the mean ± S.D. of duplicate samples. *P* values of <0,05 were considered significant.

## Supporting Information

Figure S1 **Kinetics of heat-induced binding of HUVEC HSF1 to an HSP70 HSE** ***in vitro*** **.** HUVECs were subjected to heat shock at 43°C or left untreated (Control). After 20 and 40 min at 43°C (HS) or at the indicated times during recovery at 37°C (recovery), whole-cell extracts were analyzed for HSF DNA-binding activity by EMSA in a 4% polyacrylamide gel using an HSP70 HSE ideal probe [26]. Position of HSF-DNA binding complex (HSF), constitutive HSE binding activity (CHBA) and non-specific protein-DNA interactions (NS) are shown.
(TIF)

Figure S2 **Analysis of** ***in vitro*** **and** ***in vivo*** **binding of HSF1 to the COX-2 promoter in heat-stressed human colon carcinoma cells.** **(A)** HCT116 cells were subjected to heat shock at 43°C or left untreated (control). After 40 min at 43°C (HS) or at the indicated times during recovery at 37°C (recovery), nuclear extracts were analyzed for HSF DNA-binding activity by EMSA in a 3% polyacrylamide gel using the probe described in Fig. 5A. Position of HSF-DNA binding complex (HSF) and non-specific protein-DNA interactions (NS) are shown. **(B)** Specificity of HSF1-DNA binding complexes. Nuclear extracts from HCT116 cells subjected to heat shock at 43°C for 40 min were preincubated with different dilutions of anti-HSF1 polyclonal antibodies for 15 min before electromobility supershift assay. Position of HSF and NS are indicated as in **A**. **(C,D)** HCT116 cells were subjected to heat shock at 43°C or left untreated (C). After 40 min at 43°C (HS) or at the indicated times during recovery at 37°C (recovery), recruitment of HSF1 to the COX-2 and HSP70 promoters was analyzed by ChIP assay. **(C)** ChIP-enriched DNAs using preimmune serum (IP NS IgG) or anti-HSF1 serum (IP anti-HSF1), as well as input DNAs (INPUT) were prepared, and DNA fragments of the COX-2 gene (−2629 to −2420) and HSP70 gene (−262 to −70) were amplified by PCR. **(D)** Quantification of ChIP assay shown in **(C)**. Samples from at least three independent experiments were analyzed by real time PCR. Relative promoter occupancy is expressed as fold induction of control arbitrarily set to a value of 1. Error bars indicate ± S.D.
(TIF)

Figure S3 **Stable suppression of HSF1 by RNA interference reduces heat-induced COX-2 mRNA expression in HCT116 cells.** pHCT116-retro and pHCT116-HSFi cells were subjected to heat shock at 43°C or left untreated (control). After 40 min at 43°C (HS) or at the indicated times during recovery at 37°C (recovery), total RNA was extracted and levels of COX-2 and HSP70 were analyzed by real-time PCR. Relative quantities of COX-2 and HSP70 RNAs were normalized to β-actin. All reactions were made in duplicates using samples derived from at least three biological repeats. Error bars indicate ± S.D. Fold induction was calculated by comparing the induction of the indicated genes in the treated samples to the relative control, which was arbitrarily set to 1. **(B)** In parallel samples whole-cell extracts were analyzed for levels of HSF1 and β-actin proteins by Western blot. Arrow indicates the position of the low-mobility phosphorylated HSF1 isoform.
(TIF)

Figure S4 **Effect of exposure to febrile temperatures on COX-2 protein levels and PGE_2_ production in endothelial cells**. HUVECs were either kept at 37°C (control) or were incubated at 40 and 41°C as described in Material and Methods. At 8 hours after continuous exposure at the indicated temperature whole-cell extracts were analyzed for levels of COX-2 and β-actin proteins by Western blot **(A)**, and PGE_2_ production in the culture supernatants was determined by ELISA **(B)**. Data in **B** represent the mean ± S.D. of triplicate samples from a representative experiment of two with similar results. * = P<0.05.
(TIF)

## References

[1] Morimoto RI, Santoro MG (1998). Stress-inducible responses and heat shock proteins: new pharmacologic targets for cytoprotection.. Nat Biotechnol.

[2] Morimoto RI (1998). Regulation of the heat shock transcriptional response: cross talk between a family of heat shock factors, molecular chaperones, and negative regulators.. Genes Dev.

[3] Morimoto RI (2008). Proteotoxic stress and inducible chaperone networks in neurodegenerative disease and aging.. Genes Dev.

[4] Anckar J, Sistonen L (2007). Heat shock factor 1 as a coordinator of stress and developmental pathways.. Adv Exp Med Biol.

[5] Sandqvist A, Bjork JK, Akerfelt M, Chitikova Z, Grichine A (2009). Heterotrimerization of heat-shock factors 1 and 2 provides a transcriptional switch in response to distinct stimuli.. Mol Biol Cell.

[6] Holmberg CI, Tran SE, Eriksson JE, Sistonen L (2002). Multisite phosphorylation provides sophisticated regulation of transcription factors.. Trends Biochem Sci.

[7] Abravaya K, Phillips B, Morimoto RI (1991). Attenuation of the heat shock response in HeLa cells is mediated by the release of bound heat shock transcription factor and is modulated by changes in growth and in heat shock temperatures.. Genes Dev.

[8] Westerheide SD, Anckar J, Stevens SM, Sistonen L, Morimoto RI (2009). Stress-inducible regulation of heat shock factor 1 by the deacetylase SIRT1.. Science.

[9] Trinklein ND, Murray JI, Hartman SJ, Botstein D, Myers RM (2004). The role of heat shock transcription factor 1 in the genome-wide regulation of the mammalian heat shock response.. Mol Biol Cell.

[10] Rossi A, Trotta E, Brandi R, Arisi I, Coccia M (2010). AIRAP, a new human heat shock gene regulated by heat shock factor 1.. J Biol Chem.

[11] Maity TK, Henry MM, Tulapurkar ME, Shah NG, Hasday JD (2011). Distinct, gene-specific effect of heat shock on heat shock factor-1 recruitment and gene expression of CXC chemokine genes.. Cytokine.

[12] Egan K, FitzGerald GA (2006). Eicosanoids and the vascular endothelium.. Handb Exp Pharmacol.

[13] Ricciotti E, FitzGerald GA (2011). Prostaglandins and inflammation.. Arterioscler Thromb Vasc Biol.

[14] Lu Y, Wahl LM (2005). Oxidative stress augments the production of matrix metalloproteinase-1, cyclooxygenase-2, and prostaglandin E2 through enhancement of NF-kappa B activity in lipopolysaccharide-activated human primary monocytes.. J Immunol.

[15] Caughey GE, Cleland LG, Gamble JR, James MJ (2001). Up-regulation of endothelial cyclooxygenase-2 and prostanoid synthesis by platelets. Role of thromboxane A2.. J Biol Chem.

[16] Shin YK, Park JS, Kim HS, Jun HJ, Kim GE (2005). Radiosensitivity enhancement by celecoxib, a cyclooxygenase (COX)-2 selective inhibitor, via COX-2-dependent cell cycle regulation on human cancer cells expressing differential COX-2 levels.. Cancer Res.

[17] de Gregorio R, Iniguez MA, Fresno M, Alemany S (2001). Cot kinase induces cyclooxygenase-2 expression in T cells through activation of the nuclear factor of activated T cells.. J Biol Chem.

[18] Chen Q, Fisher DT, Clancy KA, Gauguet JM, Wang WC (2006). Fever-range thermal stress promotes lymphocyte trafficking across high endothelial venules via an interleukin 6 trans-signaling mechanism.. Nat Immunol.

[19] Mbonye UR, Wada M, Rieke CJ, Tang HY, Dewitt DL (2006). The 19-amino acid cassette of cyclooxygenase-2 mediates entry of the protein into the endoplasmic reticulum-associated degradation system.. J Biol Chem.

[20] Parfenova H, Parfenov VN, Shlopov BV, Levine V, Falkos S (2001). Dynamics of nuclear localization sites for COX-2 in vascular endothelial cells.. Am J Physiol Cell Physiol.

[21] Tanabe T, Tohnai N (2002). Cyclooxygenase isozymes and their gene structures and expression.. Prostaglandins Other Lipid Mediat.

[22] Kang YJ, Mbonye UR, DeLong CJ, Wada M, Smith WL (2007). Regulation of intracellular cyclooxygenase levels by gene transcription and protein degradation.. Prog Lipid Res.

[23] Mbonye UR, Song I (2009). Posttranscriptional and posttranslational determinants of cyclooxygenase expression.. BMB Rep.

[24] Heinemeyer T, Wingender E, Reuter I, Hermjakob H, Kel AE (1998). Databases on transcriptional regulation: TRANSFAC, TRRD and COMPEL.. Nucleic Acids Res.

[25] Ray S, Lu Y, Kaufmann SH, Gustafson WC, Karp JE (2004). Genomic mechanisms of p210BCR-ABL signaling: induction of heat shock protein 70 through the GATA response element confers resistance to paclitaxel-induced apoptosis.. J Biol Chem.

[26] Mosser DD, Theodorakis NG, Morimoto RI (1988). Coordinate changes in heat shock element-binding activity and HSP70 gene transcription rates in human cells.. Mol Cell Biol.

[27] Kline MP, Morimoto RI (1997). Repression of the heat shock factor 1 transcriptional activation domain is modulated by constitutive phosphorylation.. Mol Cell Biol.

[28] Singh IS, Viscardi RM, Kalvakolanu I, Calderwood S, Hasday JD (2000). Inhibition of tumor necrosis factor-alpha transcription in macrophages exposed to febrile range temperature. A possible role for heat shock factor-1 as a negative transcriptional regulator.. J Biol Chem.

[29] Gothard LQ, Ruffner ME, Woodward JG, Park-Sarge OK, Sarge KD (2003). Lowered temperature set point for activation of the cellular stress response in T-lymphocytes.. J Biol Chem.

[30] Murapa P, Gandhapudi S, Skaggs HS, Sarge KD, Woodward JG (2007). Physiological fever temperature induces a protective stress response in T lymphocytes mediated by heat shock factor-1 (HSF1).. J Immunol.

[31] Hasday JD, Singh IS (2000). Fever and the heat shock response: distinct, partially overlapping processes.. Cell Stress Chaperones.

[32] Marnett LJ (2000). Cyclooxygenase mechanisms.. Curr Opin Chem Biol.

[33] Sawaoka H, Dixon DA, Oates JA, Boutaud O (2003). Tristetraprolin binds to the 3′-untranslated region of cyclooxygenase-2 mRNA. A polyadenylation variant in a cancer cell line lacks the binding site.. J Biol Chem.

[34] Pang L, Nie M, Corbett L, Knox AJ (2003). Cyclooxygenase-2 expression by nonsteroidal anti-inflammatory drugs in human airway smooth muscle cells: role of peroxisome proliferator-activated receptors.. J Immunol.

[35] Fujimoto M, Nakai A (2010). The heat shock factor family and adaptation to proteotoxic stress.. FEBS J.

[36] Zhao YH, Zhou M, Liu H, Ding Y, Khong HT (2009). Upregulation of lactate dehydrogenase A by ErbB2 through heat shock factor 1 promotes breast cancer cell glycolysis and growth.. Oncogene.

[37] Heintzman ND, Hon GC, Hawkins RD, Kheradpour P, Stark A (2009). Histone modifications at human enhancers reflect global cell-type-specific gene expression.. Nature.

[38] Robertson AG, Bilenky M, Tam A, Zhao Y, Zeng T (2008). Genome-wide relationship between histone H3 lysine 4 mono- and tri-methylation and transcription factor binding.. Genome Res.

[39] Guertin MJ, Lis JT (2010). Chromatin landscape dictates HSF binding to target DNA elements.. PLoS Genet.

[40] Sakurai H, Enoki Y (2010). Novel aspects of heat shock factors: DNA recognition, chromatin modulation and gene expression.. FEBS J.

[41] Calderwood SK, Bornstein B, Farnum EK, Stevenson MA (1989). Heat shock stimulates the release of arachidonic acid and the synthesis of prostaglandins and leukotriene B4 in mammalian cells.. J Cell Physiol.

[42] Arnaud C, Joyeux-Faure M, Godin-Ribuot D, Ribuot C (2003). COX-2: an in vivo evidence of its participation in heat stress-induced myocardial preconditioning.. Cardiovasc Res.

[43] Hill JM, Lukiw WJ, Gebhardt BM, Higaki S, Loutsch JM (2001). Gene expression analyzed by microarrays in HSV-1 latent mouse trigeminal ganglion following heat stress.. Virus Genes.

[44] Evers LH, Bhavsar D, Mailander P (2010). The biology of burn injury.. Exp Dermatol.

[45] Rao W, Deng ZS, Liu J (2010). A review of hyperthermia combined with radiotherapy/chemotherapy on malignant tumors.. Crit Rev Biomed Eng.

[46] Jia D, Liu J (2010). Current devices for high-performance whole-body hyperthermia therapy.. Expert Rev Med Devices.

[47] Blatteis CM, Li S, Li Z, Feleder C, Perlik V (2005). Cytokines, PGE2 and endotoxic fever: a re-assessment.. Prostaglandins Other Lipid Mediat.

[48] Shinkawa T, Tan K, Fujimoto M, Hayashida N, Yamamoto K (2011). Heat shock factor 2 is required for maintaining proteostasis against febrile-range thermal stress and polyglutamine aggregation.. Mol Biol Cell.

[49] Jaattela M (1999). Heat shock proteins as cellular lifeguards.. Ann Med.

[50] Parsell DA, Lindquist S (1993). The function of heat-shock proteins in stress tolerance: degradation and reactivation of damaged proteins.. Annu Rev Genet.

[51] Dobson CM (2003). Protein folding and misfolding.. Nature.

[52] Xie Y, Chen C, Stevenson MA, Auron PE, Calderwood SK (2002). Heat shock factor 1 represses transcription of the IL-1beta gene through physical interaction with the nuclear factor of interleukin 6.. J Biol Chem.

[53] Inouye S, Fujimoto M, Nakamura T, Takaki E, Hayashida N (2007). Heat shock transcription factor 1 opens chromatin structure of interleukin-6 promoter to facilitate binding of an activator or a repressor.. J Biol Chem.

[54] Singh IS, Gupta A, Nagarsekar A, Cooper Z, Manka C (2008). Heat shock co-activates interleukin-8 transcription.. Am J Respir Cell Mol Biol.

[55] Rossi A, Ciafre S, Balsamo M, Pierimarchi P, Santoro MG (2006). Targeting the heat shock factor 1 by RNA interference: a potent tool to enhance hyperthermochemotherapy efficacy in cervical cancer.. Cancer Res.

[56] Rossi A, Elia G, Santoro MG (1997). Inhibition of nuclear factor kappa B by prostaglandin A1: an effect associated with heat shock transcription factor activation.. Proc Natl Acad Sci U S A.

[57] Rossi A, Kapahi P, Natoli G, Takahashi T, Chen Y (2000). Anti-inflammatory cyclopentenone prostaglandins are direct inhibitors of IkappaB kinase.. Nature.

