# Correction: Regulation of Cyclooxygenase-2 Expression by Heat: A Novel Aspect of Heat Shock Factor 1 Function in Human Cells

**DOI:** 10.1371/annotation/51e012bb-b04f-4100-990d-b59d5de45ba4

**Published:** 2013-02-28

**Authors:** Antonio Rossi, Marta Coccia, Edoardo Trotta, Mara Angelini, M. Gabriella Santoro

The beta-actin bands in the HCT116 and Jurkat panels of Figure 1A were duplicated in error, the authors are therefore providing a corrected version of Figure 1: 

**Figure:**
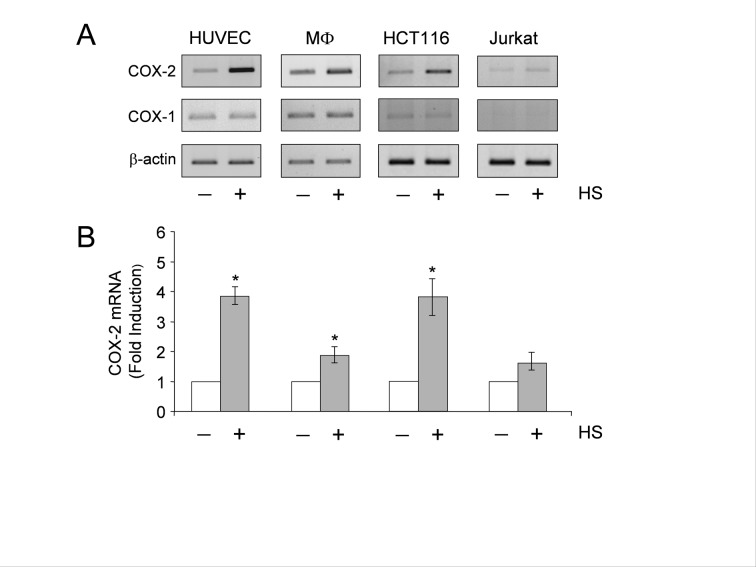


. This error does not affect the conclusions of the article. 

