# Supplementary figures and images for: Regulation of Cyclooxygenase-2 Expression by Heat: A Novel Aspect of Heat Shock Factor 1 Function in Human Cells
Source: PLoS One. 2012 Feb 8;7(2):e31304. doi: 10.1371/journal.pone.0031304 (PMC3275557; doi:10.1371/journal.pone.0031304)

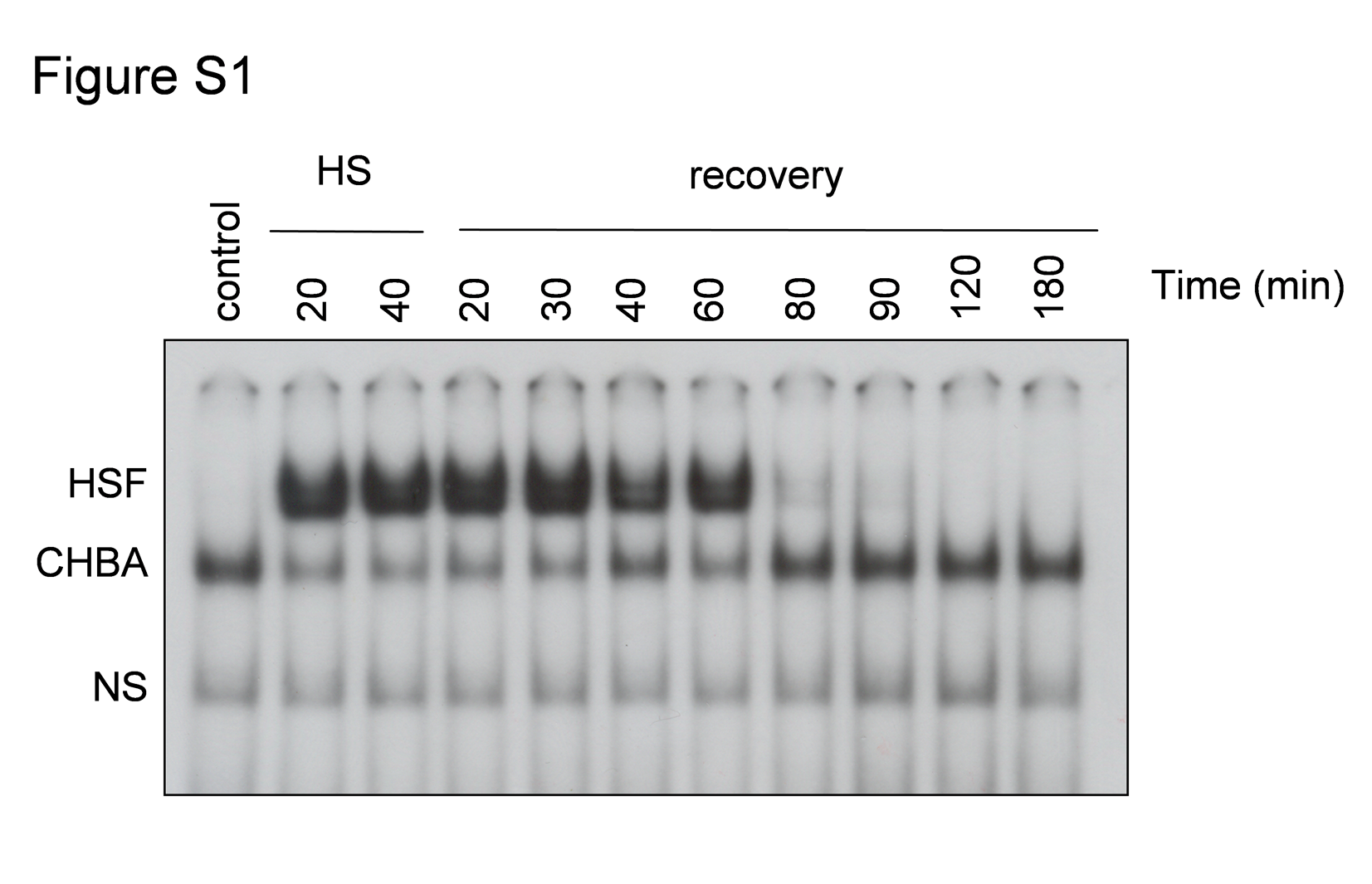

Supplement: Figure S1 — Kinetics of heat-induced binding of HUVEC HSF1 to an HSP70 HSE in vitro . HUVECs were subjected to heat shock at 43°C or left untreated (Control). After 20 and 40 min at 43°C (HS) or at the indicated times during recovery at 37°C (recovery), whole-cell extracts were analyzed for HSF DNA-binding activity by EMSA in a 4% polyacrylamide gel using an HSP70 HSE ideal probe [26]. Position of HSF-DNA binding complex (HSF), constitutive HSE binding activity (CHBA) and non-specific protein-DNA interactions (NS) are shown. (TIF) [file pone.0031304.s001.tif]

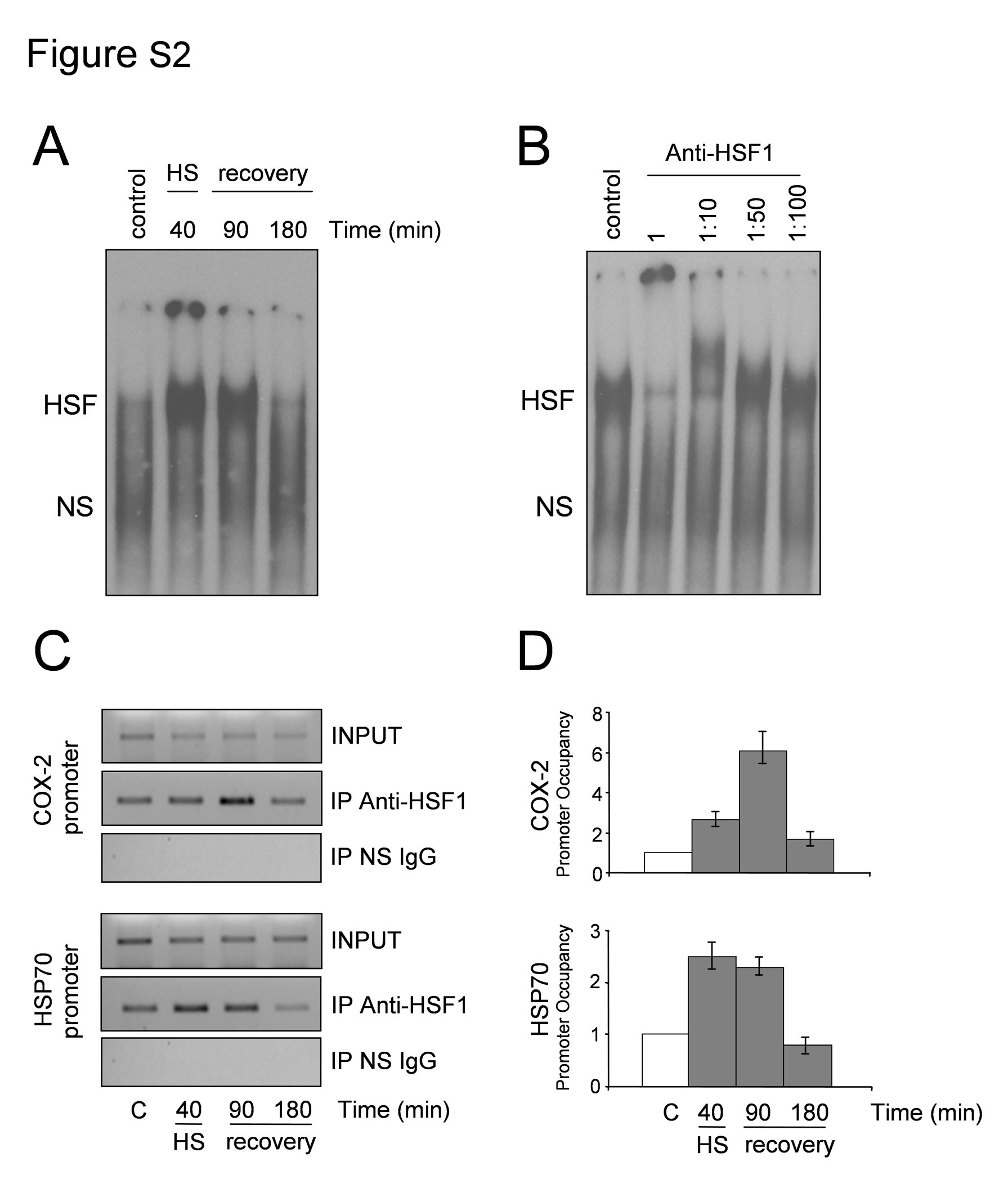

Supplement: Figure S2 — Analysis of in vitro and in vivo binding of HSF1 to the COX-2 promoter in heat-stressed human colon carcinoma cells. (A) HCT116 cells were subjected to heat shock at 43°C or left untreated (control). After 40 min at 43°C (HS) or at the indicated times during recovery at 37°C (recovery), nuclear extracts were analyzed for HSF DNA-binding activity by EMSA in a 3% polyacrylamide gel using the probe described in Fig. 5A. Position of HSF-DNA binding complex (HSF) and non-specific protein-DNA interactions (NS) are shown. (B) Specificity of HSF1-DNA binding complexes. Nuclear extracts from HCT116 cells subjected to heat shock at 43°C for 40 min were preincubated with different dilutions of anti-HSF1 polyclonal antibodies for 15 min before electromobility supershift assay. Position of HSF and NS are indicated as in A. (C,D) HCT116 cells were subjected to heat shock at 43°C or left untreated (C). After 40 min at 43°C (HS) or at the indicated times during recovery at 37°C (recovery), recruitment of HSF1 to the COX-2 and HSP70 promoters was analyzed by ChIP assay. (C) ChIP-enriched DNAs using preimmune serum (IP NS IgG) or anti-HSF1 serum (IP anti-HSF1), as well as input DNAs (INPUT) were prepared, and DNA fragments of the COX-2 gene (−2629 to −2420) and HSP70 gene (−262 to −70) were amplified by PCR. (D) Quantification of ChIP assay shown in (C). Samples from at least three independent experiments were analyzed by real time PCR. Relative promoter occupancy is expressed as fold induction of control arbitrarily set to a value of 1. Error bars indicate ± S.D. (TIF) [file pone.0031304.s002.tif]

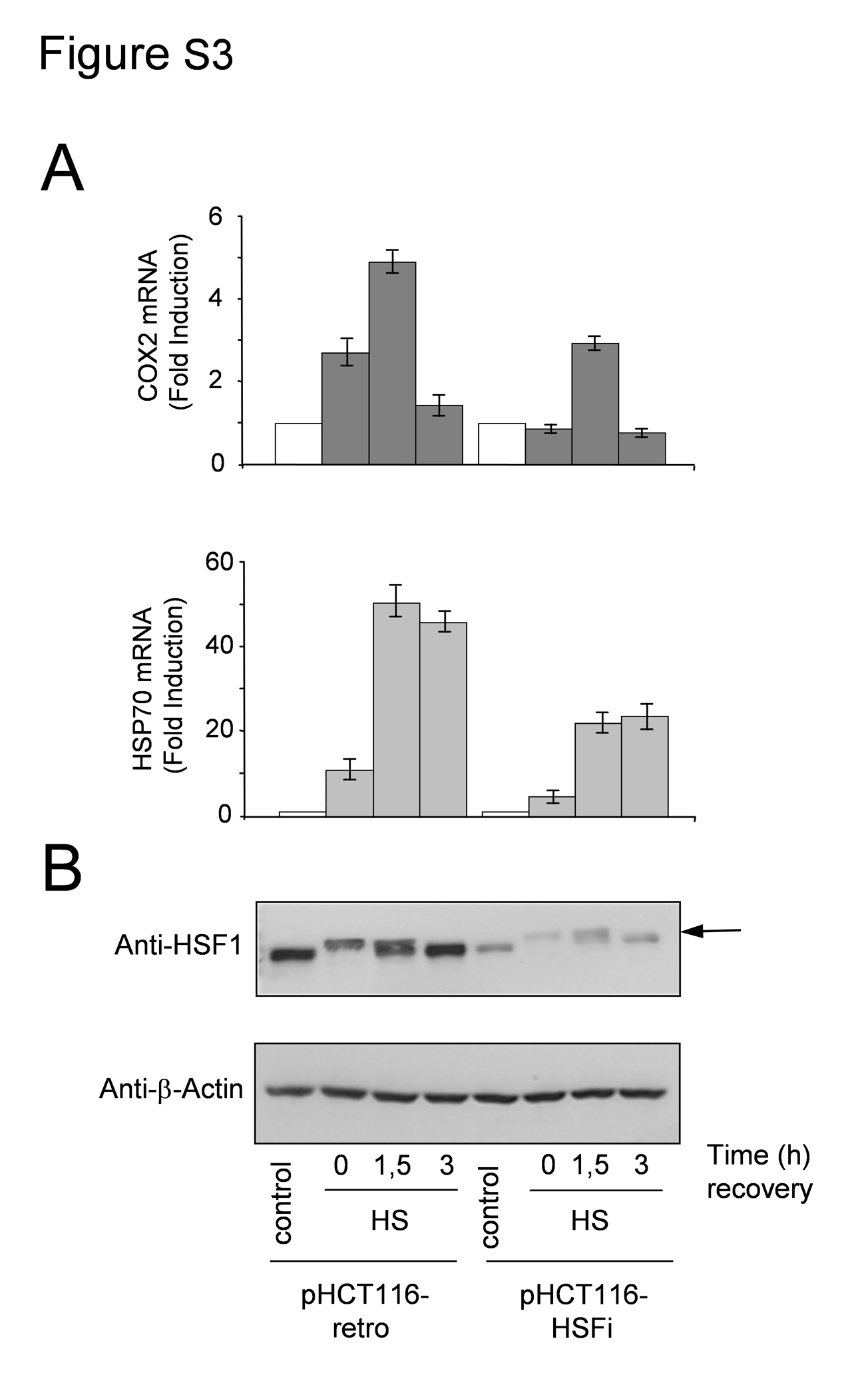

Supplement: Figure S3 — Stable suppression of HSF1 by RNA interference reduces heat-induced COX-2 mRNA expression in HCT116 cells. pHCT116-retro and pHCT116-HSFi cells were subjected to heat shock at 43°C or left untreated (control). After 40 min at 43°C (HS) or at the indicated times during recovery at 37°C (recovery), total RNA was extracted and levels of COX-2 and HSP70 were analyzed by real-time PCR. Relative quantities of COX-2 and HSP70 RNAs were normalized to β-actin. All reactions were made in duplicates using samples derived from at least three biological repeats. Error bars indicate ± S.D. Fold induction was calculated by comparing the induction of the indicated genes in the treated samples to the relative control, which was arbitrarily set to 1. (B) In parallel samples whole-cell extracts were analyzed for levels of HSF1 and β-actin proteins by Western blot. Arrow indicates the position of the low-mobility phosphorylated HSF1 isoform. (TIF) [file pone.0031304.s003.tif]

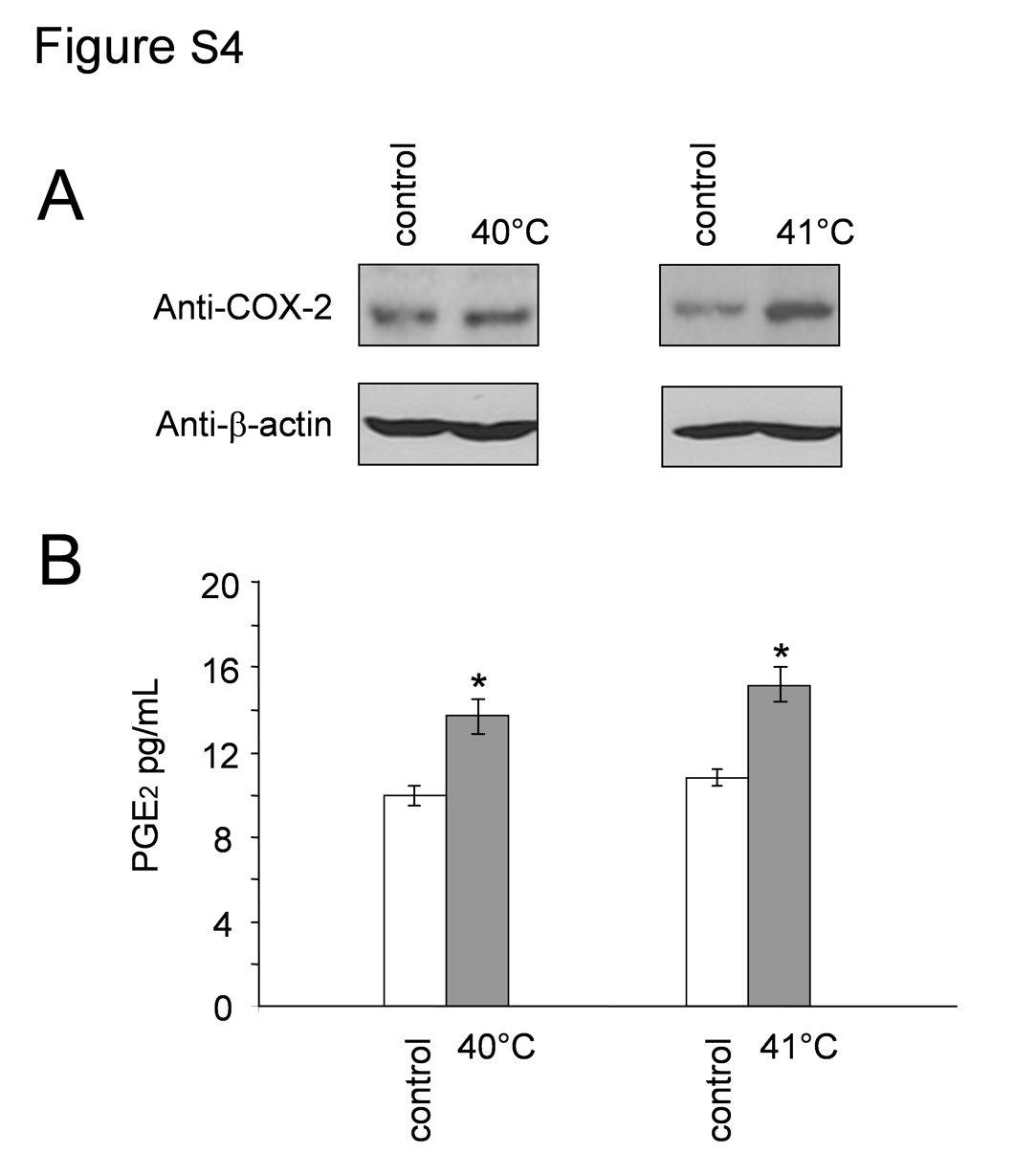

Supplement: Figure S4 — Effect of exposure to febrile temperatures on COX-2 protein levels and PGE2 production in endothelial cells. HUVECs were either kept at 37°C (control) or were incubated at 40 and 41°C as described in Material and Methods. At 8 hours after continuous exposure at the indicated temperature whole-cell extracts were analyzed for levels of COX-2 and β-actin proteins by Western blot (A), and PGE2 production in the culture supernatants was determined by ELISA (B). Data in B represent the mean ± S.D. of triplicate samples from a representative experiment of two with similar results. * = P<0.05. (TIF) [file pone.0031304.s004.tif]
